# Mass Spectrometric Blood Metabogram: Acquisition, Characterization, and Prospects for Application

**DOI:** 10.3390/ijms24021736

**Published:** 2023-01-15

**Authors:** Petr G. Lokhov, Elena E. Balashova, Oxana P. Trifonova, Dmitry L. Maslov, Anatoly I. Grigoriev, Elena A. Ponomarenko, Alexander I. Archakov

**Affiliations:** 1 Institute of Biomedical Chemistry, 10 Building 8, Pogodinskaya Street, 119121 Moscow, Russia; 2 Institute of Biomedical Problems, Russian Federation State Scientific Research Center, Russian Academy of Sciences, 123007 Moscow, Russia

**Keywords:** metabolomics, blood, metabolite set enrichment analysis, blood metabolome composition, diagnostics, metabogram, mass spectrometry, clinical blood tests, personalized metabolomics

## Abstract

In metabolomics, many metabolites are measured simultaneously in a single run. Such analytical performance opens up prospects for clinical laboratory diagnostics. In this work, a mass spectrometric metabogram was developed as a simplified and clinically applicable way of measuring the blood plasma metabolome. To develop the metabogram, blood plasma samples from healthy male volunteers (n = 48) of approximately the same age, direct infusion mass spectrometry (DIMS) of the low molecular fraction of samples, and principal component analysis (PCA) of the mass spectra were used. The seven components of the metabogram defined by PCA, which cover ~70% of blood plasma metabolome variability, were characterized using a metabolite set enrichment analysis (MSEA) and clinical test results of participating volunteers. It has been established that the components of the metabogram are functionally related groups of the blood metabolome associated with regulation, lipid–carbohydrate, and lipid–amine blood components, eicosanoids, lipid intake into the organism, and liver function thereby providing a lot of clinically relevant information. Therefore, metabogram provides the possibility to apply the metabolomics performance in the clinic. The features of the metabogram are also discussed in comparison with the thin-layer chromatography and with the analysis of blood metabolome by liquid chromatography combined with mass spectrometry.

## 1. Introduction

Following genomics and proteomics, the results of metabolomics for medical purposes are very promising [1,2,3,4,5,6,7,8]. Metabolomics, with its ability to detect big sets of metabolites, allows the use of a set of measured metabolites that differ from the control values to form multivariate characteristics—barcodes, signatures, molecular assembles, metabolic fingerprints, etc. The Metabolomics Society has noted that the study of metabolism at the global or ‘omics’ level is a rapidly growing field that can profoundly impact medical practice. Today, physicians use only a tiny part of the information in the metabolome. They usually measure only a limited set of substances in the blood to assess health and disease. The Metabolomics Society has declared that ‘*the narrow range of chemical analyses in current use by the medical community today will be replaced in the future by analyses that reveal far more comprehensive metabolic signatures. These signatures are expected to describe global biochemical aberrations that reflect patterns of variance in states of wellness, more accurately describe specific diseases and their progression, and greatly aid in differential diagnosis*’ [9].

Indeed, the metabolic data accumulated in databases over the last decades demonstrate that the blood metabolome is a collector of measurable low molecular signatures for the diversity of diseases and abnormal organism conditions [10]. All this indicates that the introduction of personalized metabolomics, i.e., the use of high-throughput measurements of large sets of low-molecular-weight substances in biosamples for clinical purposes of a particular person, has long been actual [3,4,5,6,7,8]. However, despite the evident potential, the only company that has made metabolomic tests available is Metabolon, Inc. In 2018, they announced the availability of the Meta UDx™ test for the diagnosis of rare and undiagnosed diseases in children and adults [11].

One of the reasons why metabolomics tests have not been widely used in clinical practice is that the main application of metabolomics is science and metabolomics methods were designed for scientific research, are laborious and time-consuming, and require complex data processing by scientists qualified in metabolomics. Of cause, such methods can be applied to the analysis of biosamples for a single person, but these will be single-subject scientific studies of the same level of complexity. Thus, with a variety of metabolomics methods, their use in the clinic is not visible.

**Figure 1:**
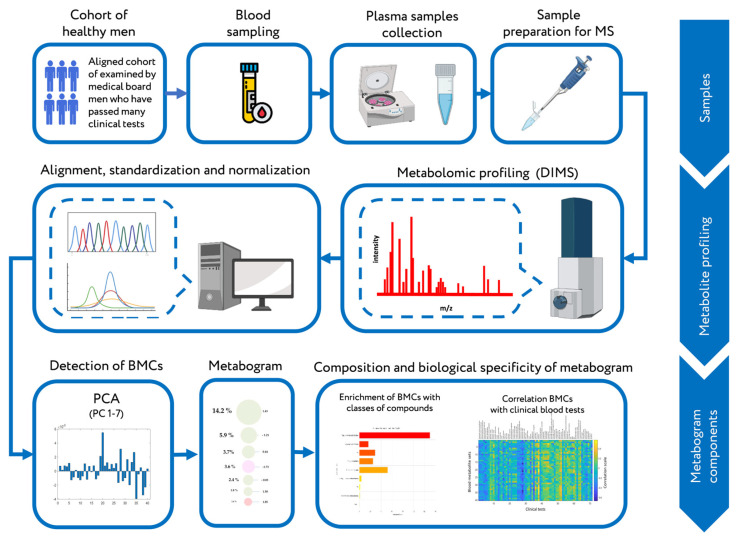
The workflow used to develop and characterize mass spectrometric blood metabogram. Blood plasma samples were taken from healthy people and after sample preparation, the mass spectra of blood metabolites were obtained by direct infusion mass spectrometry (DIMS). After data preprocessing (alignment, standardization, and normalization), the resulting lists of mass peaks were analyzed using principal component analysis (PCA) to identify the blood metabolome components (BMCs). The first seven BMCs formed the metabogram components. To characterize each metabogram component, their composition was determined by identifying the chemical classes with which they are enriched by applying a metabolite set enrichment analysis (MSEA). To clarify the biological specificity of the metabogram components, clinical blood tests were used.

This study proposes a concept for solving a central problem in the application of personalized metabolomics in the clinic by developing a metabogram—a mass-spectrometry-based measurement of the blood metabolome with clinical test-specific parameters for speed of execution, reproducibility of results, speed of data processing and ease of perception of the result. The study workflow is shown in Figure 1.

## 2. Results

### 2.1. Subjects and Mass Spectrometry of Blood Plasma Metabolome

The astronaut candidate selection program made it possible to form a cohort for this study, which consists of healthy men of a similar age after full medical examination, and clinical blood tests which corresponded to the norm or negligibly deviated from the normal values (Table S1). The volunteer’s blood samples were processed according to the workflow shown in Figure 1. Direct infusion mass spectrometry (DIMS) was used for obtaining mass spectra of the low-molecular-weight fraction of blood plasma samples. On average, 9973 peaks were detected in the mass spectrum (s.d. ± 345).

### 2.2. PCA of Mass Spectrometry Data of Blood Plasma Samples

**Figure 2:**
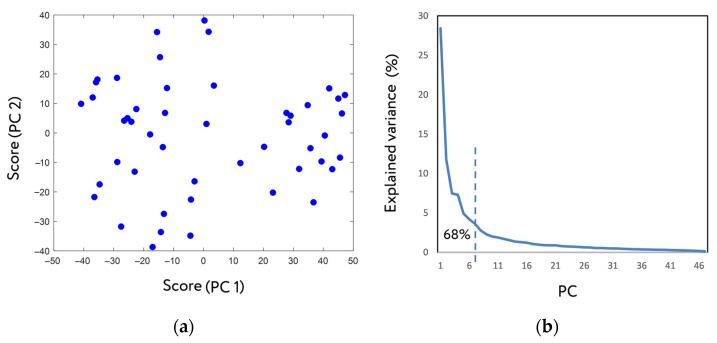
Principal component analysis (PCA) of the mass spectrometry data of the blood plasma samples of healthy male volunteers. (**a**) PCA score plot of the first two principal components of the data. (**b**) The plot of variance explained by each principal component (PC). The first seven PCs explain 68% of blood metabolome variance.

Principal component analysis (PCA) of the aligned, standardized, and normalized mass peak lists showed the homogeneity of the metabolic data of the samples and the absence of outliers (Figure 2a), which justified their further use for defining blood metabolome components (BMCs).

### 2.3. Composition of Blood Metabolome Components

Of all the BMCs formed from loadings of the principal components, the first seven, explaining 68% of the total variance present in the blood metabolome (Figure 2b), were selected to determine their composition. For this, MetaboAnalyst was used to conduct a metabolite set enrichment analysis (MSEA) to identify chemical classes enriching selected BMCs (Table 1).

The MSEA showed that the main blood metabolome components are mainly enriched by lipids and on less degree by carbohydrates and amino acids. This is quite understandable because most blood metabolites are lipids [14]. Thus, the proposed mass-spectrometry-based visual presentation of the main groups of the blood metabolome was called a mass spectrometric metabogram.

Despite some overlap of metabolite classes among BMCs, since the same metabolites may be simultaneously involved in various biologic processes, specific features in the composition of the BMCs are evident. BMC 1 can be called a ‘regulatory’ component since it consists of a lot of steroids, to which many regulatory substances are related. BMC 2 can be called the ‘phospholipid-carbohydrate’ component, due to phospholipids together with carbohydrates are characteristic of it.

In BMC 3, MSEA failed to identify the dominant class of metabolites. BMC 4 is similar to component 2 in terms of the presence of phospholipids. However, instead of carbohydrates, amino acids appear in it. It may be assumed that this component of the blood metabolome reflects the associated variability in the metabolism of phospholipids and blood amines, which probably is determined by identical factors.

BMC 5 is characterized by the presence of different metabolite groups (eicosanoids, unsaturated fatty acids, retinoids, and dicarboxylic acids) but does not contain steroids and phospholipids, except for their lysoforms. This component is enriched with such eicosanoids as prostaglandins and leukotrienes, which makes it possible to suggest that lysoforms in this component are connected with them. Lysophospholipids are formed under certain stimuli that cause the synthesis of prostaglandins and leukotrienes in the cell [15].

BMC 6 is enriched with bile acids, retinoids, and diacylglycerols. It is known that bile acids affect the production of diacylglycerols [16] and the absorption of fat-soluble substances in the intestine, including retinoids. This may be reflected in this blood metabolome component.

For chemical classes characteristic of BMC 7, a common feature can be distinguished—the relation to the liver (lysophospholipids [17,18,19,20], glycerophosphoglycerophosphates (PGP) [21], C19 steroids [22], amino acids [23]). This fact suggests that the variability of the blood metabolome described by this BMC is associated with liver function.

**Figure 3:**
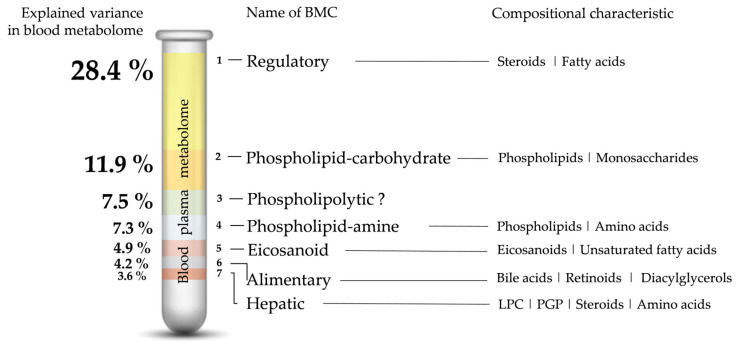
The main blood metabolome components (BMCs) and the main chemical classes that contribute to them. The first seven components are shown, each explaining at least 3% of the variance in the blood plasma metabolome and 68% altogether. The percentage shows the variance explained by BMC. Gender, age, and biological rhythms (for example, circadian rhythm) contributing to metabolome variability are not included (only men of the same age in the study cohort). PC, phosphatidylcholines; LPC, Lysophosphatidylcholines; PGP, glycerophosphoglycerophosphates.

Figure 3 schematically represents the description of the blood plasma metabolome based on the main BMCs, which explain the metabolome variance of more than 3%.

### 2.4. Drawing a Metabogram

**Figure 4:**
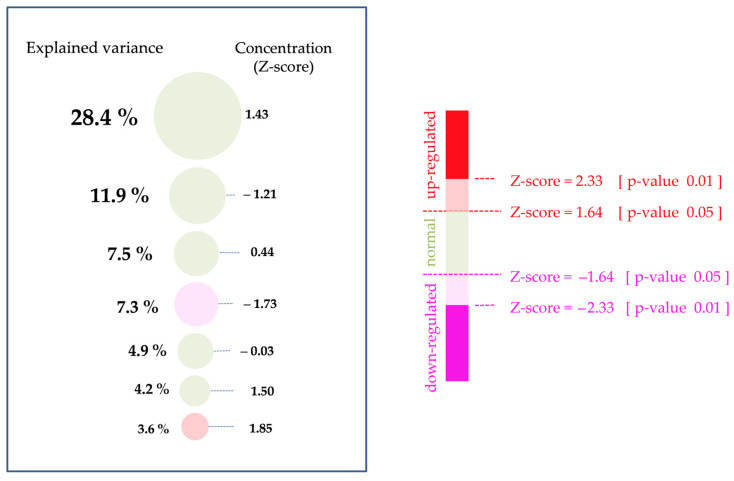
Mass spectrometric blood metabogram. The percentage shows the variance explained by each metabogram component. Gender, age, and biological rhythms (for example, circadian rhythm) contributing to metabolome variability are not included (only men of the same age in the study cohort). The Z-score value is a measure of the metabogram component (from −1.64 to +1.64 is the normal range). ‘Up-’ and ‘down-regulation’ correspond to higher and lower Z-score values, respectively.

According to the idea of creating a simplified metabolomic blood test suitable for the clinic, only the first seven BMCs, whose contribution to blood metabolome variance exceeds 3%, were considered to form the mass spectrometric metabogram of the blood (Figure 4).

### 2.5. Correlation of the Metabogram Components with Clinical Blood Tests

The metabogram components can be characterized not only by a specific composition but also by their relation to biological processes in the organism. Clinical blood tests can help with this. Such tests are usually used to check the functioning of the organism; that is, they are objective monitors of the processes that take place in the blood or are reflected there. The correlation analysis was used to identify the connection between the metabogram components (i.e., the mean Z-score of metabolites in each BMC that form the metabogram) and the clinical blood tests following the Cohen scale [24]: correlation coefficients of 0.30–0.49 are considered as ‘moderate’ connection, whereas 0.50–1.00—‘strong’ connection.

**Figure 5:**
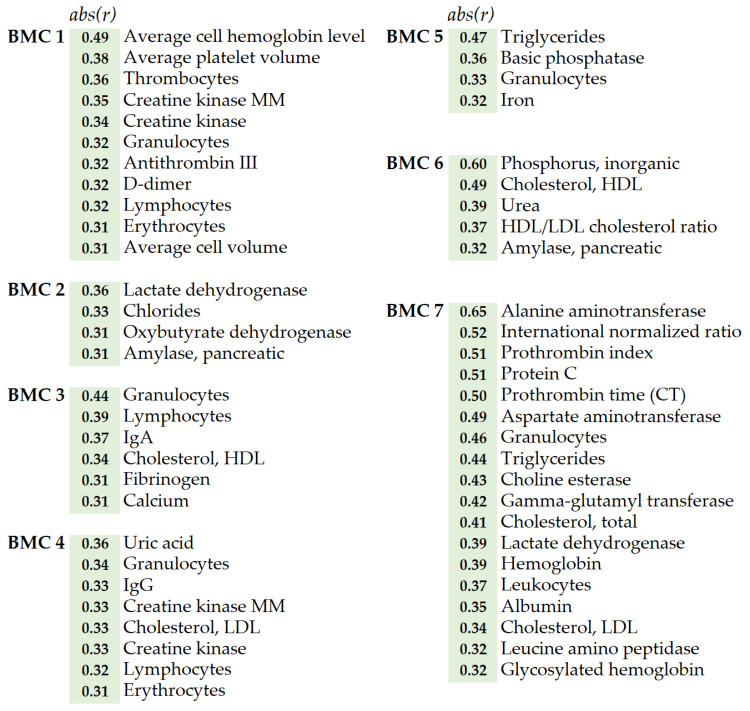
Correlation of the blood metabogram components with the clinical blood tests. Only moderate and strong correlations are presented. Full data are presented in Figure S1. *Abs*(*r)*—absolute value of correlation coefficient *r* (demonstrates the strength of the connection between clinical test and BMC). BMC—blood metabolome component.

Correlation analysis showed that metabogram components are moderately and strongly associated with clinical blood tests (Figure 5). At the same time, some components highly correlate with some clinical blood tests and do not correlate with others (Figure S1), which indicates their biological specificity and selective relationship with processes reflected in the blood. For example, BMC 6 correlates with inorganic phosphorus (correlation coefficient *r* = −0.60), with which BMC 7 does not correlate (*r* = 0.06) (Figure S1b). In turn, BMC 7 is strongly correlated with alanine aminotransferase (*r* = 0.65), with which BMC 6 is not correlated (*r* = −0.01) (Figure S1a).

BMC 1, called ‘regulatory’ because it mainly contains steroids, is associated with several blood tests. It is known, that steroids affect the red blood cell level, hemoglobin level, and blood coagulation parameters [25] that are reflected in correlation with erythrocytes, average cell hemoglobin level, thrombocytes, antithrombin, and D-dimer clinical tests. Additionally, steroids affect the level of creatine kinase [26], which is also consistent with clinical blood tests.

The connection of BMC 2, called the ‘phospholipid-carbohydrate’ component, with clinical tests is explicit. This BMC correlates with clinical tests related to carbohydrate metabolism (lactate dehydrogenase, oxybutyrate dehydrogenase, pancreatic amylase, and fructose amine tests). The correlating level of chlorides, reflecting the acidification of the blood, is also connected with carbohydrate metabolism. The phospholipid component of this BMC is reflected by the HDL/LDL cholesterol ratio test (phospholipids are not directly presented in the used clinical tests).

Clinical tests associated with BMC 3 allowed hypothesizing that metabolome variability in this component is driven by phospholipase activity. Main substances contributing to metabogram components, such as phospholipids, lysophospholipids, fatty acids, and oxo fatty acids (e.g., eicosanoids), are closely related to phospholipases. Phospholipases release fatty acids from phospholipids, which in turn are converted into oxoform by the action of oxygenases. BMC 3 correlation with calcium test supports this suggestion. There are calcium-dependent phospholipases [27]. For example, lipoprotein-associated phospholipase A2 (PLA2) is calcium-dependent and hydrolyses the glycerophospholipids of lipoproteins and cell membranes to produce lysoPC and free fatty acids [28] which are oxidized by cyclooxygenase or lipoxygenase to produce eicosanoids. Eicosanoids include thromboxanes and prostacyclins which affect blood clotting, possibly reflected by BMC 3 correlation with the fibrinogen test [29]. PLA2 is directly connected with platelet-activating factor (PAF) [30], which affects leukocytes and this is also reflected in clinical tests (correlation with the level of granulocytes). Correlation with lymphocytes as well as with IgA may be explained by eicosanoids’ influence on their level [31,32].

Thus, although the action of phospholipases extends to many classes of lipids presented on the metabogram, they are proteins and are not represented on it. This explains why MSEA was unable to identify the main characteristic class of substances for BMC 3, although the variability of the blood metabolome, explained by this component, ranks third in importance. All this allows to presumably call this BMC ‘phospholipolytic’.

BMC 4, in which a linkage of phospholipids and amino acids was seen, the correlation with creatine kinase tests can be considered as confirming sign for this, due to creatine is an amino acid. The phospholipid component of this BMC is reflected by the LDL cholesterol test (amino acids and phospholipids are not directly represented in the used clinical tests). Thus, clinical tests confirm the given name ‘phospholipid-amine’ for this BMC.

BMC 5 according to clinical tests is a metabogram component highly associated with triglycerides. However, triglycerides are not directly presented in this component, which is not surprising. The sample preparation protocol with methanol chosen in this study leads to a decrease in the detection of triglycerides [33]. However, the triglyceride level is associated with eicosanoids which are well-detectable and well-expressed in BMC 5 [34].

BMC 6 is enriched with bile acids, retinoids, and diacylglycerols and correlates with inorganic phosphorus in the blood, and, in less degree, with cholesterol and urea. It is known that phosphate concentration in blood is dependent on dietary intake. The major source of retinoids is from the diet too. A significant proportion of the lipids in the body are obtained from the diet [35], and fats must be first emulsified by bile salts [36] to form a mixture of tri-, di- and monoglycerides together with the other fat-soluble contents of the diet (e.g., the fat-soluble vitamins and cholesterols) [35,37]. One of the main sources of diacylglycerols enriching this metabolite set is a diet too. One of the two physiological causes influencing the urea level is dietary proteins. Therefore, this BMC can be considered closely related to food consumption and can be called the ‘alimentary’ component of metabogram.

Clinical tests have revealed the relationship between the last component of the metabogram (BMC 7) with the liver. Aspartate aminotransferase, alanine aminotransferase, and gamma-glutamyl transferase, usually used to assess liver status, correlate with BMC 7 with correlation coefficients of 0.49, 0.65, and 0.42, respectively. Moreover, this BMC is characterized by the presence of lysoPCs. Blood plasma lysoPCs are mainly secreted from the liver [17,18,19,20]. Albumin (which test estimates liver function), protein C (produced by the liver [38]), and cholesterol (liver breaks down cholesterol) levels are also affected by the liver. Therefore, clinical tests and composition show that the last component of the metabogram is the ‘hepatic’ component.

Thus, the composition of the metabogram components, which cover significant variance of the blood metabolome, is generally consistent with clinical tests, that supports their biological specificity.

### 2.6. Metabogram Reproducibility and Variability

To assess the reproducibility and variability of the metabogram, as a criterion for its applicability as a bioanalytical test, the CV was calculated. The metabogram has reproducibility and variability significantly better than DIMS and LC-MS (Table 2). The obvious reason for this is that metabogram components are formed by covariant metabolites, values for which are unified (translated to Z-score scale) and averaged, which significantly improve the technical reproducibility and decrease biological variability of metabogram components. Thus, the metabogram meets the requirements for analytical tests in terms of reproducibility (CV less than 15% [39]) and is an acceptable method for introducing metabolomics into the clinic.

## 3. Discussion

The introduction of high-throughput methods of analysis, widely used in omics sciences, including metabolomics, is an expected stage in the development of clinical laboratory diagnostics [42]. A similar view on metabolomics methods is shared by the Metabolomics Society [9]. However, the ways of implementing metabolomics methods may be different. In light of the introduction of omics technologies into medicine, it is interesting to look at single-subject (N-of-1) studies, where they are used to analyze the biomaterial of a single person. Such technologies can be considered the basis for personalized medicine. Therefore, in personalized medicine, the iPOP (Integrated Personal Omics Profiling) direction is known, where the concept of implementation is to obtain various omics data for a person [43]. That is, due to the integrated use of omics technologies, additional knowledge about the person is obtained. In other words, the implementation of iPOP is based on increasing the value of the results at the expense of the complexity of the analysis.

In this work, which is related to personalized metabolomics, a new approach is implemented. The authors of this work believe that the widespread implementation of omics tests in clinics can be facilitated. To do this, an N-of-1 study must acquire the features of a clinical test, making it attractive for medical applications, namely in time of execution, compliance with requirements, simplicity in data processing, and ease of interpretation of the results. To do this the metabogram was designed using well-known, widespread, and proven methods, such as DIMS, PCA, and MSEA. Moreover, the last two methods are involved only in the creation of the metabogram and are no longer used in its further routine application. The bottleneck of any N-of-1 metabolomics study—the analysis of the data obtained—has been scientifically soundly simplified. Therefore, this work on the development of a metabogram can be considered a proof of concept for a new approach in personalized metabolomics.

For reliable detection of new biological data, all other features of studied biological objects should be aligned as much as possible. This was achieved in this study by using healthy, examined, and selected volunteers of the same sex and approximately the same age.

The choice of DIMS for revealing blood metabolome groups is not accidental. In addition to good reproducibility, DIMS provides the metabolomics data of the sample -without the additional distortion introduced by the separation methods, be it liquid or gas chromatography [44]. The use of chromatography could complicate the identification of blood metabolome components. Coeluted substances from the column, due to ion suppression, can influence each other intensities, and thus the chromatography can affect the PCA of mass spectrum data [45].

PCA allowed identifying of the groups of mass spectrometry peaks of the blood metabolites that formed the metabogram. PCA is a popular technique for analyzing datasets containing a high number of features per observation, increasing data interpretability by reducing dimensionality while preserving the maximum amount of information. This is achieved by transforming the dataset into a new coordinate system that shows closely related features. Thus, the use of PCA to develop a metabogram as a reduced representation of the blood metabolome that preserves the most of information, with the possibility of identifying biologically relevant groups to describe the components of the metabogram, was reasonable.

The enrichment analysis showed that the main BMCs are predominantly enriched with lipids, and the lipid composition is specific to each BMC. The representation of the main BMCs by lipids is entirely expected. Lipid-soluble molecules are a major part of chemical substances in the blood metabolome [14,46]. Lipids and lipid-like substances represent a significant and chemically diverse fraction of metabolome (>80,000 lipid molecules exist in humans, and more than 20,000 of them are found in the blood), which play an essential role in living systems. Various functional groups of lipids make them versatile machines serving as cellular barriers (various phospho- and glycolipids [47]), membrane matrices (cholesterols), signaling agents (ceramide, sphingosine), and energy reservoirs (triglycerides) [46,47,48].

The biological specificity of BMCs was supported by correlation analysis with the clinical blood tests. Such tests are usually used to assess health status; that is, they are objective monitors of the specific processes that take place in the organism and are reflected in blood. The correlation analysis showed that metabogram components are associated with almost all clinical blood tests (Figure S1), which was not surprising. The metabolome is a level of organization of biological systems directly related to global biochemical phenotype [49]. It is noteworthy that some metabogram components highly correlate with some clinical blood tests and do not correlate with others indicating their specificity concerning certain biological processes reflected in the blood.

Thus, although the main BMCs, which form metabogram, demonstrate some overlap since the same metabolites participate in several biological processes at once, the metabogram components have compositional and biological specificity. This fact justifies their use to determine the state of the body by checking the regulatory, lipid-carbohydrate, lipid-amine, alimentary, eicosanoid, and hepatic components of the blood metabolome reflected in the metabogram. In total, almost 70% of the global biochemical phenotype of the organism is estimated. Therefore, a metabogram can potentially be considered a personalized metabolomics tool [50].

To understand the analytical advantages of the metabogram, it is worth comparing it with TLC used to analyze the lipid composition of the blood and with blood metabolome analysis by LC-MS (Table 3). TLC uses silica gel with a uniform and small particle size, providing a good separation. For example, the main classes of lipids in clinical samples can be obtained using one-dimensional TLC in a single chromatographic run.

A comparison of methods shows that the metabogram is a relatively simple, but precise and informative method well-suited for clinical application. A distinctive feature is that the metabogram consists of metabolites grouped based on covariance, which makes the metabogram innovative compared to other methods. Metabogram is closely related to the reflection of the functioning of organism systems. Moreover, being a simplified metabolomics analysis of blood, the metabogram may be easier introduced in the clinic than LC-MS.

Regarding the weaknesses of the metabogram, BMCs underlying the metabogram are formed from experimental data and therefore depend on the used protocols. For example, the sample preparation protocol with methanol chosen in this study leads to a decrease in the detection of triglycerides [33]. Despite the BMCs are a reflection of processes in the body, and this makes them versatile, the dependence on the measurement protocols makes them quite biased, and therefore they may have slight differences in different studies until the reference BMCs are not deposited in the widely used public database.

In this light, the variety of ionization methods used in metabolomics should also be noted. Different ionization methods lead to the detection of different sets of metabolites [51], and this should be reflected in the metabogram. In this work, ESI was used as the most widely used in metabolomics. DIMS with ESI in positive ion charge detection mode has been used successfully in the past, and the analysis protocol used gives a mass spectrum with more intense peaks than in negative ion charge detection mode [52]. The feasibility of using two ionization options simultaneously to build a metabogram has yet to be clarified since routine operations are duplicated, which is somewhat inconsistent with the idea underlying the metabogram, namely, to make metabolome analysis as simple as a clinical test.

The next issue is associated with used metabolite databases, where the redundancy of compound names exists. Together with the high combinatorial capabilities of lipids (e.g., phospholipids have a variety of fatty acid chains, resulting in many compounds with the same molecular weight), it makes the MSEA less reliable.

Although the mass spectrometer was equipped with ESI source, which is considered to be soft, some metabolites are susceptible to in-source fragmentation. This is especially true for phospholipids fragmenting into lysoforms and fatty acids, which affects the composition of BMCs, where such compounds are present. This source of the mass spectrometry data variation is not crucial if mass spectra are obtained in the same laboratory under the same conditions, i.e., at the same voltage in an electrospray ionization source. However, such a source of mass spectrometry data variation complicates the comparison of metabograms obtained in different laboratories.

The future research directions will include the study of the influence of gender, age, circadian and other rhythms on metabogram, as well as the identification of the relationship between metabogram and genome, microbiome, and diet, the influence of which on the blood metabolome is well known [53]. The second line of further research is related to clinical goals. The metabogram components correlate with numerous clinical tests (Figure 5) and reflect the main processes in the blood metabolome. Moreover, blood lipids are considered biomarkers of different diseases among which all major types of cancer, atherosclerosis, diabetes, hepatitis, and cirrhosis of the liver, chronic liver failure, bronchial asthma, and even Alzheimer’s disease, and depressive states [54]. Therefore, the study of the diagnostic potential of metabogram is of particular interest, especially in light of the possibility of introducing metabograms into clinical practice as simplified and reproducible blood metabolome analysis.

## 4. Materials and Methods

### 4.1. Subjects

Forty-eight male normosthenic subjects (age 20–36, average 25.0, s.d. ±4.0) were examined at the Institute of Biomedical Problems (Russian Federation State Scientific Research Center, Russian Academy of Sciences, Moscow, Russia) by the medical board, which specializes in space biology and medicine [55]. All participants were approved for space-related simulations and experiments according to their health characteristics. They were HIV, viral hepatitis B, and C negative and had no previous history of cancer. The volunteers’ routine biochemical and blood parameters were measured using standard automatic analyzers (Table 4). Most parameter values fit the regular ranges known in clinical laboratory practice (see Table S1). All participants signed the informed consent to participate in this study. Human-related procedures were performed at the Institute of Biomedical Problems according to the guidelines of the local ethical committee.

### 4.2. Blood Sample Preparation

Venous blood was collected from the volunteers into EDTA Vacutainer plasma tubes (BD, USA) and was processed according to the manufacturer’s instructions [56]. The resultant blood plasma was stored at −80 °C until analysis. The analyzed samples were subjected to one freeze/thaw cycle. Plasma (10 μL) was mixed with 10 μL of water (LiChrosolv; Merck KGaA, Darmstadt, Germany) and 80 μL of methanol (Fluka, Munich, Germany). After incubation at room temperature for 10 min, the samples were centrifuged at 13,000× *g* (Centrifuge 5804R; Eppendorf AG, Hamburg, Germany) for 15 min. The supernatant was then transferred to clean plastic Eppendorf™ tubes, and fifty volumes of methanol containing 0.1% formic acid (Fluka) were added to each tube. The resulting solutions were subjected to mass spectrometry analysis.

### 4.3. Mass Spectrometry

Samples were analyzed by a hybrid quadrupole time-of-flight mass spectrometer (maXis Impact, Bruker Daltonics, Billerica, MA, USA) equipped with an electrospray ionization (ESI) source (drying gas temperature (nitrogen)—180 °C; drying gas flow rate—4 L/min; capillary voltage—4000 V; focusing voltage—500 V; electrospray pressure—0.3 bar; low-pressure funnel RF voltage—90V; high-pressure funnel RF voltage—120 V). The mass spectrometer was set up to prioritize the detection of ions with a mass-to-charge ratio (*m/z*) ranging from 45 to 900, with a mass accuracy of 1–3 parts per million (ppm). The spectra were recorded in the positive ion charge detection mode. The samples were injected into the ESI source using a glass syringe (Hamilton Bonaduz AG, Bonaduz, Switzerland) connected to a syringe injection pump (KD Scientific, Holliston, MA, USA). The rate of sample flow to the ionization source was 180 µL/h. Mass spectra were obtained using DataAnalysis version 4.1 (Bruker Daltonics) to summarize one-minute signals.

### 4.4. Mass Spectra Processing

Peak detection, recalibration, and peak intensity calculation of mass spectra were carried out automatically by DataAnalysis software. Masses of compounds were determined from the mass spectrum peaks obtained using the following parameters: peak width, 2; signal-to-noise ratio, 1; relative and absolute threshold intensity, 0.01% and 100, respectively. For the recalibration of all the peak *m/z* values, the internal standard losartan (*m/z* 423.169) was used.

### 4.5. Mass Lists Processing

Standardization of mass peak intensities was performed as described previously [57] by dividing the intensity by the standardization value, which was calculated for each peak separately as follows: the 50 Da range (started 25 Da before and ended 25 Da after the *m/z* of the mass peak) was selected; all peaks inside the range were sorted in descending order according to their intensities; the intensity of the 150th peak was selected as the standardization value. Standardized intensities improved the further used correlation analysis due to the correction of ion suppression of peak intensities [57]. Standardized mass lists were normalized by applying the *normalize* function (brings the sum of the intensities of the peaks in the spectrum to 1) of the Matlab program (version R2019a; MathWorks, Natick, MA, USA). Alignment of the *m/z* values of the mass peaks between different mass spectra was performed as described previously [58]. The alignment algorithm used was previously specially developed and tested for the high-resolution mass spectra of blood metabolites obtained by DIMS and implemented as an iterative process based on the detection of correlation of mass spectrometry peak patterns.

### 4.6. Detection of Blood Metabolome Components (BMCs)

Aligned, standardized, and normalized mass lists with *m/z* values (n = 2864) present in all mass spectra were analyzed using PCA (*pca* function of the Matlab program). *m/z* values relating to 5% of the highest and lowest coefficients (loadings) of each principal component were used to compile BMCs. Each principal component was associated with one BMC with the same number. The principal components are ranked according to their contribution to the explanation of the data. Therefore, the BMCs describe blood metabolome in the same order. The first seven BMCs, which explain the most pronounced variations in the blood, were further processed.

### 4.7. Composition of BMCs

The sets of *m/z* values corresponding to the first seven BMCs were separately submitted to the metabolite search engine, MassTRIX (http://masstrix3.helmholtz-muenchen.de; accessed on 15 September 2022), with the following parameters: scan mode, positive ionization (corrected for H+, Na+, K+ adducts); max. error, 0.005 Da; database, KEGG/HMDB/LipidMaps with isotopes; organism, Homo sapiens. The resulting lists of metabolite names were further processed by MSEA [59] to determine the composition of the analyzed BMCs. MSEA was applied using MetaboAnalyst 5.0 software (https://www.metaboanalyst.ca/MetaboAnalyst/home.xhtml; accessed on 15 September 2022) [60] with the following options: module, enrichment analysis (ORA—over-representation analysis); input type, HMDB ID; feature type: metabolites or lipids; metabolite set library, chemical structures (1072 chemical sub-class metabolite sets or lipid sets); isotopes were excluded. Enrichment analysis returns a *p*-value that evaluates whether a measured set of metabolites is represented more than expected by chance within a given chemical class (the lower *p*-value corresponds to higher over-representation (enrichment)). Thus, the resulting *p*-values showed which chemical classes of compounds enrich BMCs.

### 4.8. Metabogram Components and Their Measure

The first seven BMCs, whose contribution to blood metabolome variance exceeds 3%, were used to form metabogram components. The mean value of the Z-scores of the intensities of the mass peaks belonging to the same metabogram component was considered as the measure of this component. Converting the mass peak intensities to a Z-score allows calculating the mean intensity of the mass peaks with different intensity ranges that are represented in each metabogram component. The mean value was considered as a measure of the corresponding component of the metabogram, which is directly related to the change in the concentration of metabolites involved in the construction of the metabogram components.

### 4.9. Drawing a Metabogram

Metabogram is seven circles, called metabogram components, and circles correspond to the first seven BMCs, respectively. The size of the circle corresponds to explained by BMC the variance in blood plasma metabolome. The color of circles corresponds to the Z-score value of BMC with color coding following ranges: below −2.33, from −2.33 to −1.64, from −1.64 to 1.64 (norm), from 1.64 to 2.33, above 2.33. Since the metabogram is based on precise data, the exact explained variance and Z-scores are also presented to the right and left of the circles, respectively. Increased and decreased Z-scores correspond to a proportional change in the concentration of metabolites in the metabogram component and allow to speak about the up- or down-regulation of the associated biological processes.

### 4.10. Metabogram Correlation with Clinical Tests

The Spearman’s correlation between the measure of metabogram components (which indicates the change in the concentration of the metabolites in corresponding BMC) and the clinical blood test results for each person was calculated using the *corr* function of the Matlab program. The Cohen scale [24] identified a correlation of 0.5 as being on the cusp of ‘medium/moderate’ (0.30–0.49) and ‘large/strong’ (0.50–1.00).

### 4.11. Metabogram Reproducibility and Variability

The reproducibility and variability of the metabogram were assessed by the coefficient of variation (CV—s.d. divided by mean value). To assess the technical reproducibility, the mass spectra were obtained 12 times for the same blood plasma sample (male, 36 years old). Blood samples from 8 individuals (4 males and 4 females, average age of 35 years old) were used to measure inter-individual biological variability. To measure the intra-individual biological variability, samples of the same 8 people were used, obtained twice with a break of three months.

The CV for the metabogram was calculated indirectly. Since a direct calculation of CV for Z-scores is not possible (due to the mean value of the Z-scores distribution being zero), the following formula was used to calculate CV for metabogram:


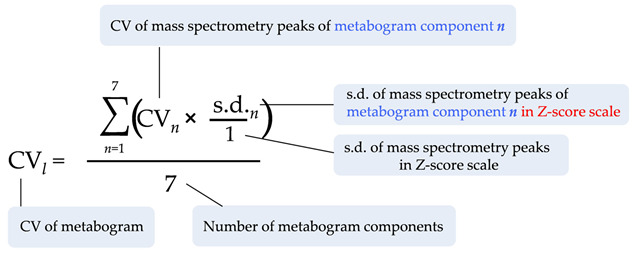


Knowing that s.d. of mass peaks intensities in the Z-score scale always is equal to 1, the s.d. for peaks in each metabogram component was calculated, and its difference from 1 directly reflects the CV improvement for metabogram components (due to CV is s.d. divided by mean and mean is always unchanged in Z-score scale). Knowing how much the CV is improved for metabogram components, the CV of mass peaks of metabogram components before converting to Z-score was changed proportionally to obtain the absolute CV value for metabogram components. The final result for the metabogram is a mean CV of all metabogram components. Reproducibility and variability data for liquid chromatography combined with mass spectrometry (LC-MS) were taken from the literature [40]. Reproducibility data for thin-layer chromatography (TLC) were taken from the literature [41]. CV value of less than 15% for technical (analytical) reproducibility was considered acceptable for the practical application of the metabogram as a bioanalytical test [39].

## 5. Conclusions

A new concept for the introduction of blood metabolome analysis into medicine has been proposed. While maintaining the omics features, the analysis of the blood metabolome was given the features of clinical tests. Using the mass spectrometric measurement of thousands of blood metabolites, a digital metabogram is created that represents the blood metabolome in a simplified, precise, and reproducible manner. Metabogram allows for checking the main processes that are reflected in the blood metabolome, providing physicians with clinically relevant information in an easily perceptible form. Based on the combination of features, the metabogram can be considered promising for a wide clinical application.

## Figures and Tables

**Table 1:** Composition of the blood metabolome components (BMCs) determined by their enrichment with chemical classes.

Chemical Class (Metabolite Group)	Fold Enrichment (*p*-Value ^1^)						
	BMC 1	BMC 2	BMC 3	BMC 4	BMC 5	BMC 6	BMC 7
Phosphatidylcholines	-	3.0 × 10^−^ ^153^	-	6.1 × 10^−2^ ^44^	-	-	-
Phosphatidylethanolamines	-	5.0 × 10^−^ ^10^	-	2.0 × 10^−^ ^19^	-	-	-
Monosaccharides	-	3.0 × 10^−6^	-	-	-	-	-
Saturated Fatty Acids	4.4 × 10^−8^	-	-	-	-	-	-
C18 steroids	7.0 × 10^−29^	-	-	-	-	-	-
C10 isoprenoids	2.1 × 10^−8^	-	-	-	-	-	-
C24 bile acids ^2^	-	-	-	-	-	1.6 × 10^−12^	
Dicarboxylic acids	0.0006	-	-	-	3.4 × 10^−4^	-	-
Unsaturated Fatty Acids	4.0 × 10^−11^	-	-	-	7.7 × 10^−5^	-	-
Lysophosphatidylcholines ^3^	-	-	-	-	2.3 × 10^−5^	-	7.2 × 10^−17^
Lysophosphatidylethanolamines ^3^	-	-	-	-	-	-	3.2 × 10^−27^
Diacylglycerols	-	-	-	-	-	9.4 × 10^−37^	7.7 × 10^−42^
Retinoids	-	-	-	-	6.0 × 10^−8^	6.0 × 10^−12^	-
Amino acids	-	-	-	4.6 × 10^−15^	8.0 × 10^−26^	-	4.2 × 10^−20^
Androstane steroids	8.3 × 10^−23^	-	-	-	-	-	4.6 × 10^−11^
C19 steroids	2.8 × 10^−35^	-	-	-	-	-	6.8 × 10^−12^
Glycerophosphoglycerophosphates	-	-	-	-	-	-	6.4 × 10^−15^
Estrane steroids	4.0 × 10^−5^	-	-	-	-	-	-
Leukotrienes	-	-	-	-	8.0 × 10^−14^	-	-
Prostaglandins	-	-	-	-	3.1 × 10^−10^	-	-

^1^ *p*-value evaluates whether a measured set of metabolites is represented in BMC more than expected by chance within a given chemical class (the lower *p*-value corresponds to higher over-representation (enrichment); *p*-value is calculated by MSEA). The blue font corresponds to the data obtained for the highest positive coefficients (loadings) of the principal component that form the BMC. The red font corresponds to the data obtained for the lowest negative coefficients (loadings) of the principal component that form the BMC. ^2^ Despite many bile acids having the same molecular weight, leading to a significant overestimation of enrichment, the molecular weights detected in BMC 6 are characteristic of bile acids (e.g., *m/z* 391.3 [12]) that retained bile acids in the table. ^3^ The used DIMS does not distinguish native blood lysophosphatidylcholines from the lysophosphatidylcholines formed due to in-source fragmentation of phosphatidylcholines [13]. The same relates to lysophosphatidylethanolamines.

**Table 2:** Comparison of technical reproducibility and biological variability between mass spectrometric metabogram, direct mass spectrometry, liquid chromatography combined with mass spectrometry, and clinical tests.

Method	Technical Reproducibility (CV ^1^)	Biological Variability (CV)	
		Interindividual	Intra-Individual ^2^
DIMS	10% (1–76) ^3^	44% (12–394)	40% (7–117)
LC-MS	25% ^4^	56% ^4^	-
TLC	2.2% (1.5–2.8) ^5^	-	-
Metabogram	1.8% (1.4–2.8)	13.6% (8.4–17.6)	10.8% (7.2–16.4)
Clinical tests ^6^	-	Biochemistry tests—31% (2–117) Hematology tests—17% (5–34) Immunology tests—38% (29–45) Hemostasis tests—15% (5–54)	-

^1^ Mean and range (in brackets) value of the coefficient of variation (CV). ^2^ Blood sampling twice from same persons with a break of three months. ^3^ Mean CV values for mass peak intensities are presented. ^4^ Data from [40]. ^5^ Data from [41]. ^6^ Data source for calculating CV in Table S1. DIMS, direct infusion mass spectrometry; LC-MS, liquid chromatography combined with mass spectrometry; TLC, thin-layer chromatography.

**Table 3:** Comparative characteristics of mass spectrometric blood metabogram, TLC of blood lipids, and LC-MS analysis of blood metabolome.

Parameter	Metabogram	TLC	LC-MS
Detected substances	Groups of lipids and some main nonlipid groups (carbohydrates and amino acids).	Main groups of lipids.	Lipids and other metabolites (metabolome). Numerous separate metabolites are identified, and their concentration is measured.
Principle of substance grouping	Functional relations (covariate substances involved in the same processes).	Structural composition	No grouping
Precision of measurement	High	Low	High
Reproducibility	High. Suitable for clinical tests (CV lower 15%)	High. Suitable for clinical tests (CV lower 15%) ^1^	Low. Technical reproducibility—CV 25% ^2^ Biological reproducibility—CV 56% ^2^
Complexity	Moderate. Does not require the identification of individual lipids.	Low	High. A method is usually used in scientific research.
Results	The concentration of several functionally related metabolite groups.	The concentration of several main lipid groups.	The concentration of thousands of individual metabolites.
Time for acquisition	Quick method	Time-consuming method	Time-consuming method
The complexity of data processing	Moderate	Low	High

^1^ Data from [41]. ^2^ Data from [40]. CV, coefficient of variation; TLC, thin-layer chromatography; LC-MS, liquid chromatography combined with mass spectrometry.

**Table 4:** The cohort blood characteristics.

Parameter	Reference Levels ^1^	Mean ± s.d.
**Biochemistry**		
Aspartate aminotransferase (IU/L)	0–37	31.2 ± 15.5
Alanine aminotransferase (IU/L)	0–42	25.9 ± 14.7
Gamma-glutamyl transferase (IU/L)	11–50	28.2 ± 25.7
Glutamate dehydrogenase (IU/L)	0–7	6.6 ± 7.7
Choline esterase (IU/L)	5300–12,900	8821 ± 1356
Basic phosphatase (IU/L)	80–306	183 ± 39
Leucine amino peptidase (IU/L)	21.0–57.6	35.5 ± 5.6
Bilirubin, total (μM)	0–17.1	13.1 ± 7.4
Bilirubin direct (μM)	0–4.30	3.91 ± 2.03
Amylase, total (IU/L)	0–220	73.3 ± 18.9
Amylase, pancreatic (IU/L)	0–115	33.0 ± 13.7
Lipase (IU/L)	0–190	91.9 ± 31.3
Lipase. Pancreatic (IU/L)	0–60	39.5 ± 11.4
Creatinine (μM)	53–115	89.8 ± 12.6
Urea (mM)	1.7–8.3	4.9 ± 1.3
Total protein (g/L)	67–87	75.6 ± 4.0
Albumin (g/L)	35–50	47.2 ± 1.93
Uric acid (μM)	200–420	342 ± 78
Glucose (mM)	4.2–6.4	5.3 ± 0.5
Fructose amine (μM)	0–285	234 ± 28
Glycosylated hemoglobin (%)	4.5–7.5	5.9 ± 0.6
Creatine kinase (IU/L)	0–190	195 ± 180
Creatine kinase MM (IU/L)	0–190	175 ± 173
Creatine kinase MB (IU/L)	0–24	20.8 ± 7.3
Lactate dehydrogenase (IU/L)	225–450	311 ± 54
Oxybutyrate dehydrogenase (IU/L)	72–182	138 ± 22
Cholesterol, total (mM)	2.8–5.2	4.6 ± 0.8
Cholesterol, HDL (mM)	>0.91	1.6 ± 0.3
Cholesterol, LDL (mM)	<4.0	2.5 ± 0.6
HDL/LDL cholesterol ratio	>0.28	0.7 ± 0.2
Triglycerides (mM)	0.55–2.30	1.0 ± 0.5
Iron (μM)	6.6–26.0	17.1 ± 6.5
Calcium (mM)	2.25–2.67	2.5 ± 0.1
Magnesium (mM)	0.7–1.05	0.98 ± 0.07
Phosphorus, inorganic (mM)	0.87–1.45	1.19 ± 0.19
Chlorides (mM)	98–106	103.6 ± 2.8
Potassium (mM)	3.5–5.1	4.0 ± 0.3
Sodium (mM)	135–145	139.7 ± 2.5
Acid phosphatase (IU/L)	0–5.4	3.0 ± 0.6
Acid phosphatase, prostatic (IU/L)	0–1.7	0.9 ± 0.3
**Hematology**		
Leukocytes (×10^9^/L)	4.0–10.0	6.3 ± 1.4
Erythrocytes (×10^12^/L)	4.0–5.7	5.3 ± 0.4
Hemoglobin (g/L)	130–173	159.7 ± 8.9
Hematocrit (%)	34.0–49.0	46.0 ± 3.5
Average cell volume	80.0–100.0	87.6 ± 4.3
Average hemoglobin content per 1 erythrocyte (pg)	27.0–35.0	30.4 ± 1.7
Average cell hemoglobin level (g/L)	300–380	348 ± 17
Thrombocytes (×10^9^/L)	100–400	240 ± 49
Lymphocytes (%)	19.0–45.0	35.3 ± 8.0
Lymphocytes (×10^9^/L)	1.2–4.0	2.2 ± 0.7
Monocytes (%)	3.0–11.0	7.3 ± 1.9
Monocytes (×10^9^/L)	0.09–0.60	0.46 ± 0.16
Granulocytes (%)	42.0–85.0	57.4 ± 8.5
Granulocytes (×10^9^/L)	2.0–5.8	3.6 ± 1.0
Interval of red blood cell distribution (%)	11.5–14.5	13.7 ± 0.6
Platelet crit (%)	0.08–1.00	0.16 ± 0.03
Average platelet volume (fL)	6.0–11.0	6.8 ± 1.4
**Immunology**		
IgA (g/L)	0.70–4.00	2.4 ± 1.1
IgM (g/L)	0.40–2.30	1.3 ± 0.49
IgG (g/L)	7.0–16.0	12.3 ± 3.6
**Hemostasis**		
Prothrombin time (CT) (s)	9.8–12.7	11.5 ± 0.6
Prothrombin index (%)	70–130	106.2 ± 12.1
International normalized ratio (units)	0.85–1.15	1.0 ± 0.1
Partial thromboplastin time (s)	26.4–37.5	38.7 ± 4.0
Fibrinogen (g/L)	1.8–3.5	2.0 ± 0.3
Thrombin time (s)	14–21	19.6 ± 0.9
Antithrombin III (%)	75–125	102.9 ± 11.7
Plasminogen (%)	75–150	102.7 ± 17.3
Antiplasmin (%)	80–120	121.8 ± 9.2
Protein C (%)	70–140	112.0 ± 23.9
D-dimer (μg/L)	Up to 550	291 ± 157

^1^ Reference levels for males are provided.

## Data Availability

The data presented in this study are available on request from the corresponding author.

## Supplementary Materials

The following are available online at https://www.mdpi.com/article/10.3390/ijms24021736/s1.
