# Supplementary material for: Mass Spectrometric Blood Metabogram: Acquisition, Characterization, and Prospects for Application
Source: Int J Mol Sci. 2023 Jan 15;24(2):1736. doi: 10.3390/ijms24021736 (PMC9861083; doi:10.3390/ijms24021736)
Supplement: Supplementary file 1 [file ijms-24-01736-s001.zip › Supplementary Materials.pdf]

Supplementary Materials

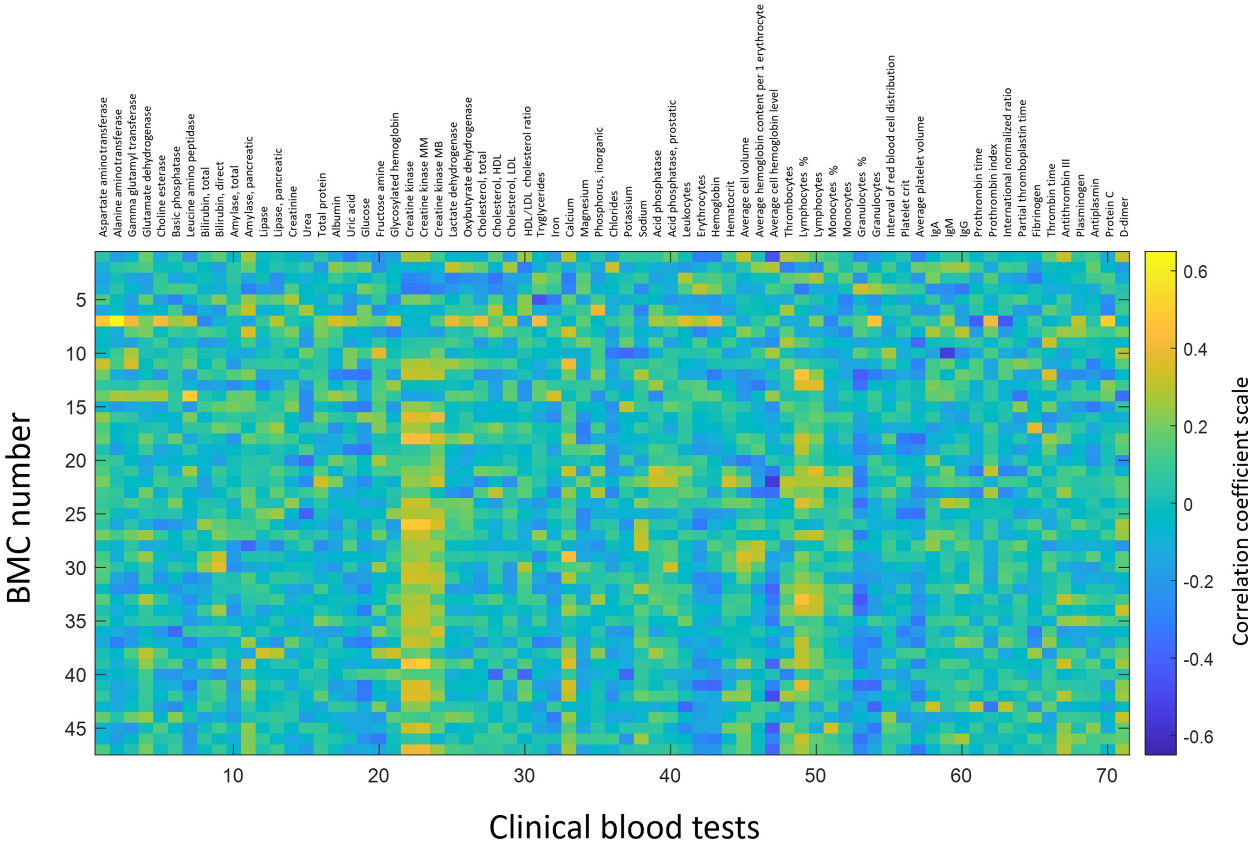

(**a**)

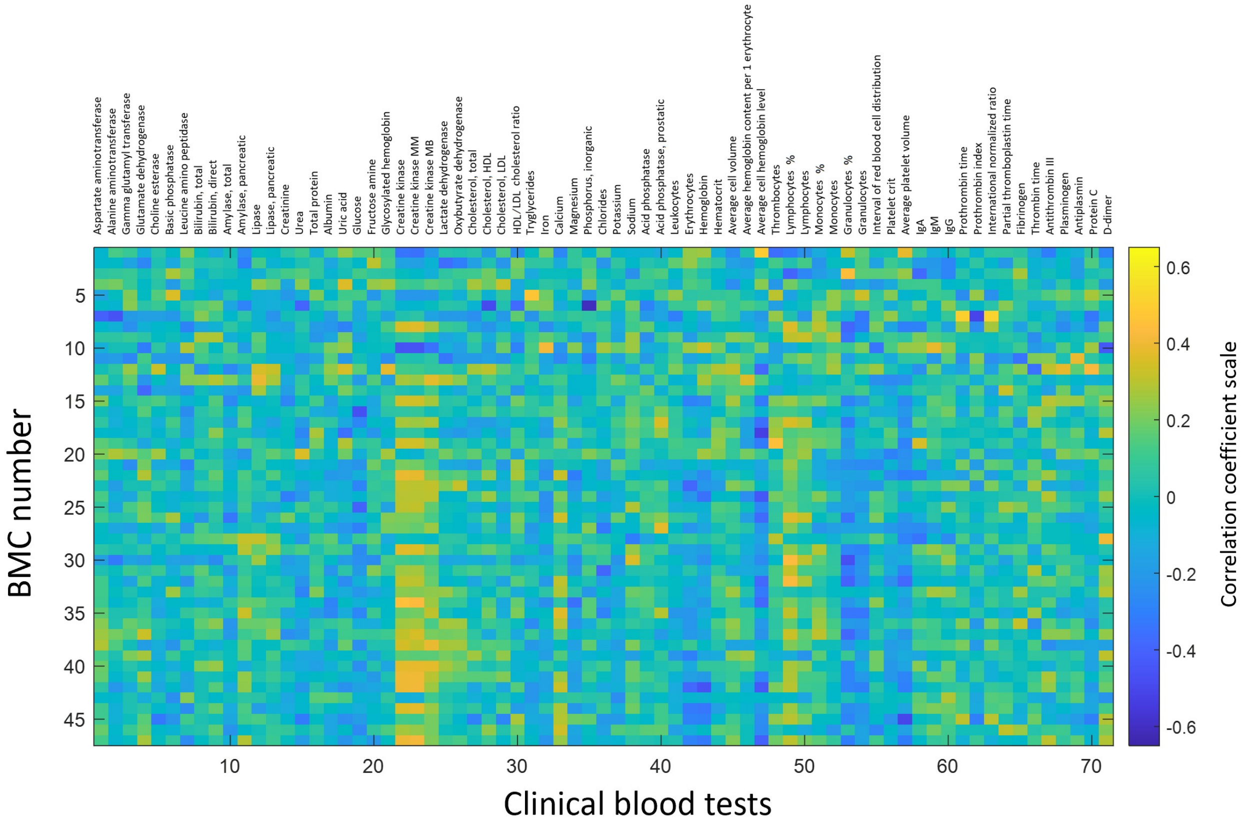

(**b**)

Figure S1. Correlation of the blood metabolome components (BMCs) with the clinical blood tests. (**a**) Data for the BMCs corresponded to the highest positive coefficients (loadings) of the principal components. (**b**) Data for the BMCs corresponded to the lowest negative coefficients (loadings) of the principal components.
